# The impact of subthreshold levels of amyloid deposition on conversion to dementia in patients with amyloid-negative amnestic mild cognitive impairment

**DOI:** 10.1186/s13195-022-01035-2

**Published:** 2022-07-11

**Authors:** Hyung-Ji Kim, Jungsu S. Oh, Jae-Sung Lim, Sunju Lee, Sungyang Jo, E-Nae Chung, Woo-Hyun Shim, Minyoung Oh, Jae Seung Kim, Jee Hoon Roh, Jae-Hong Lee, for the Alzheimer’s Disease Neuroimaging Initiative

**Affiliations:** 1 Department of Neurology, University of Ulsan College of Medicine, Asan Medical Center, Seoul, South Korea; 2 Department of Neurology, Uijeongbu Eulji Medical Center, Eulji University School of Medicine, Uijeongbu, South Korea; 3 Department of Nuclear Medicine, University of Ulsan College of Medicine, Asan Medical Center, Seoul, South Korea; 4 Health Innovation Bigdata Center, Asan Institute for Lifesciences, Department of Radiology and Research Institute of Radiology, University of Ulsan College of Medicine, Asan Medical Center, Seoul, South Korea; 5 Asan Institute for Life Sciences, Asan Medical Center, Seoul, South Korea; 6 Neuroscience Institute, Korea University College of Medicine and School of Medicine, Seoul, South Korea

**Keywords:** Amyloid, Dementia, Disease progression, Mild cognitive impairment

## Abstract

**Background:**

About 40–50% of patients with amnestic mild cognitive impairment (MCI) are found to have no significant Alzheimer’s pathology based on amyloid PET positivity. Notably, conversion to dementia in this population is known to occur much less often than in amyloid-positive MCI. However, the relationship between MCI and brain amyloid deposition remains largely unknown. Therefore, we investigated the influence of subthreshold levels of amyloid deposition on conversion to dementia in amnestic MCI patients with negative amyloid PET scans.

**Methods:**

This study was a retrospective cohort study of patients with amyloid-negative amnestic MCI who visited the memory clinic of Asan Medical Center. All participants underwent detailed neuropsychological testing, brain magnetic resonance imaging, and [^18^F]-florbetaben (FBB) positron emission tomography scan (PET). Conversion to dementia was determined by a neurologist based on a clinical interview with a detailed neuropsychological test or a decline in the Korean version of the Mini-Mental State Examination score of more than 4 points per year combined with impaired activities of daily living. Regional cortical amyloid levels were calculated, and a receiver operating characteristic (ROC) curve for conversion to dementia was obtained. To increase the reliability of the results of the study, we analyzed the Alzheimer’s Disease Neuroimaging Initiative (ADNI) dataset together.

**Results:**

During the follow-up period, 36% (39/107) of patients converted to dementia from amnestic MCI. The dementia converter group displayed increased standardized uptake value ratio (SUVR) values of FBB on PET in the bilateral temporal, parietal, posterior cingulate, occipital, and left precuneus cortices as well as increased global SUVR. Among volume of interests, the left parietal SUVR predicted conversion to dementia with the highest accuracy in the ROC analysis (area under the curve [AUC] = 0.762, *P* < 0.001). The combination of precuneus, parietal cortex, and FBB composite SUVRs also showed a higher accuracy in predicting conversion to dementia than other models (AUC = 0.763). Of the results of ADNI data, the SUVR of the left precuneus SUVR showed the highest AUC (AUC = 0.596, *P* = 0.006).

**Conclusion:**

Our findings suggest that subthreshold amyloid levels may contribute to conversion to dementia in patients with amyloid-negative amnestic MCI.

**Supplementary Information:**

The online version contains supplementary material available at 10.1186/s13195-022-01035-2.

## Background

β-Amyloid (Aβ) proteins, which are the pathological hallmark of Alzheimer’s disease (AD), begin to deposit at the pre-clinical stage of the AD continuum and are known to induce tau pathology along the disease course [1, 2]. The deposition of Aβ is a non-linear process that is initiated decades before the manifestation of clinical symptoms; hence, many questions remain about the exact point of the development of neuronal degeneration in the AD continuum as well as the best time point to initiate anti-amyloid treatment [3]. Traditionally, the concept of subthreshold levels of amyloid deposition has been used to refer to the changes in amyloid levels during the pre-clinical stages of AD [4]. However, according to previous studies, subthreshold levels of Aβ deposition are increasingly drawing attention to the importance of predicting the deposition and spreading of tau pathology as well as clinical progression along the AD continuum [4, 5]. Clinically, there had been studies indicating that both levels of amyloid and patterns of deposition were significant in cognitive functions. A previous study analyzing cerebrospinal fluid (CSF) AD biomarkers found that the dementia conversion hazard ratio (HR) of MCI with normal amyloid levels was not inferior compared to MCI with decreased CSF amyloid levels [6]. In addition, a study using a ^18^F-flutmetamol PET tracer reported that the patterns of focal amyloid deposition were associated with changes in cognitive function as well as diffuse amyloid deposition [7, 8].

In general, amnestic mild cognitive impairment (MCI) is considered to be prodromal AD and corresponds to the symptomatic stage of the AD continuum [9]. Since amyloid positron emission tomography (PET) scans enable clinicians to rule out AD, diagnostic accuracy and proper patient management have increased in recent years [10, 11]. Notably, around 40–50% of patients with amnestic MCI are known to be negative on amyloid PET [12]. In this population, conversion to dementia is known to occur in around 10% of patients within 2 years, which is lower than the conversion rate of 60–80% in amyloid-positive amnestic MCI groups [12, 13]. Importantly, many different pathologies, including vascular burden, hippocampal sclerosis, argyrophilic grain disease, and TAR-binding protein-43 (TDP-43) could be responsible for this condition [14–17]. However, its relationship to brain amyloid deposition remains largely unknown [4, 6].

Therefore, we analyzed the characteristics of patients from our prior studies who converted to dementia from amyloid-negative amnestic MCI from our prior studies [18–20]. In the converter group, we found that decreased cerebral gray matter volume associated with the visual pathway and decreased cerebellar gray matter volume in the Crus I/II area stood out as being significant compared to the non-converter group [18–20]. In this study, we hypothesized that differences in subthreshold levels of amyloid deposition affect conversion to dementia in patients with amnestic MCI who were visually negative on amyloid PET. To that end, we quantitatively analyzed the amyloid PET results of the same cohort from our prior study to reveal the impact of subthreshold amyloid deposition on conversion to dementia in amnestic MCI patients with visually negative amyloid PET scans.

## Methods and materials

### Participants

The inclusion criteria for patients were as follows: (1) age over 50 years with at least a 36-month follow-up period; (2) MCI, defined by the criteria proposed by Petersen; and (3) no evidence of amyloid deposition in the visual rating of amyloid PET scans. A total of 211 amyloid-negative MCI patients were recruited according to the aforementioned criteria from the memory clinic of Asan Medical Center from March 2013 to March 2016 [18, 19].

The diagnosis of MCI was determined based on a patient’s change in cognition, objective evidence of impairment in one or more cognitive domains (including memory, executive function, attention, language, or visuospatial skills), preservation of independence in functional abilities, and no dementia [21, 22]. Only patients with amnestic MCI were included in the study. The amnestic subtype of MCI was determined when the score was below the 16th percentile (− 1 standard deviation) for demographically matched norms in verbal (Seoul verbal learning test immediate recall, delayed recall, and recognition) or visual (Rey complex figure immediate recall, delayed recall, and recognition) memory tasks. In case of only a memory deficit, the patient was defined as a single-domain amnestic MCI. Multiple-domain amnestic MCI was defined as A-MCI patients who may have memory deficits with additional dysfunctions affecting other cognitive domains [23]. Both single- and multiple-domain amnestic MCI patients were included in the dataset [24].

According to the brain amyloid-plaque loading (BAPL) scoring system, amyloid status was defined by consensus, with BAPL1 being Aβ-negative and BAPL2 and BAPL3 being Aβ-positive. ^18^F-florbetaben (FBB) amyloid PET images were assessed visually by a consensus of board-certified nuclear medicine physicians to determine the regional cortical uptake in the frontal, lateral temporal, precuneus/posterior cingulate, and parietal regions [25, 26]. Only the patients whose ^18^F-florbetaben PET read BAPL1 were included in the dataset. Additionally, quantitative results of FBB composite scores were also considered. In cases of FBB composite scores which were defined as the average of SUVRs of the frontal, lateral temporal, parietal, anterior, and posterior cingulate and precuneus, which are known to be vulnerable to amyloid deposition in AD above 1.32, the patient was determined to be amyloid-positive and was excluded from the dataset [27, 28]. This was included in the inclusion criteria for focusing on patients with amyloid-negative specifically. From the SUVR obtained by quantitative analysis, the amyloid positivity was determined by an FBB composite score.

**Fig. 1:**
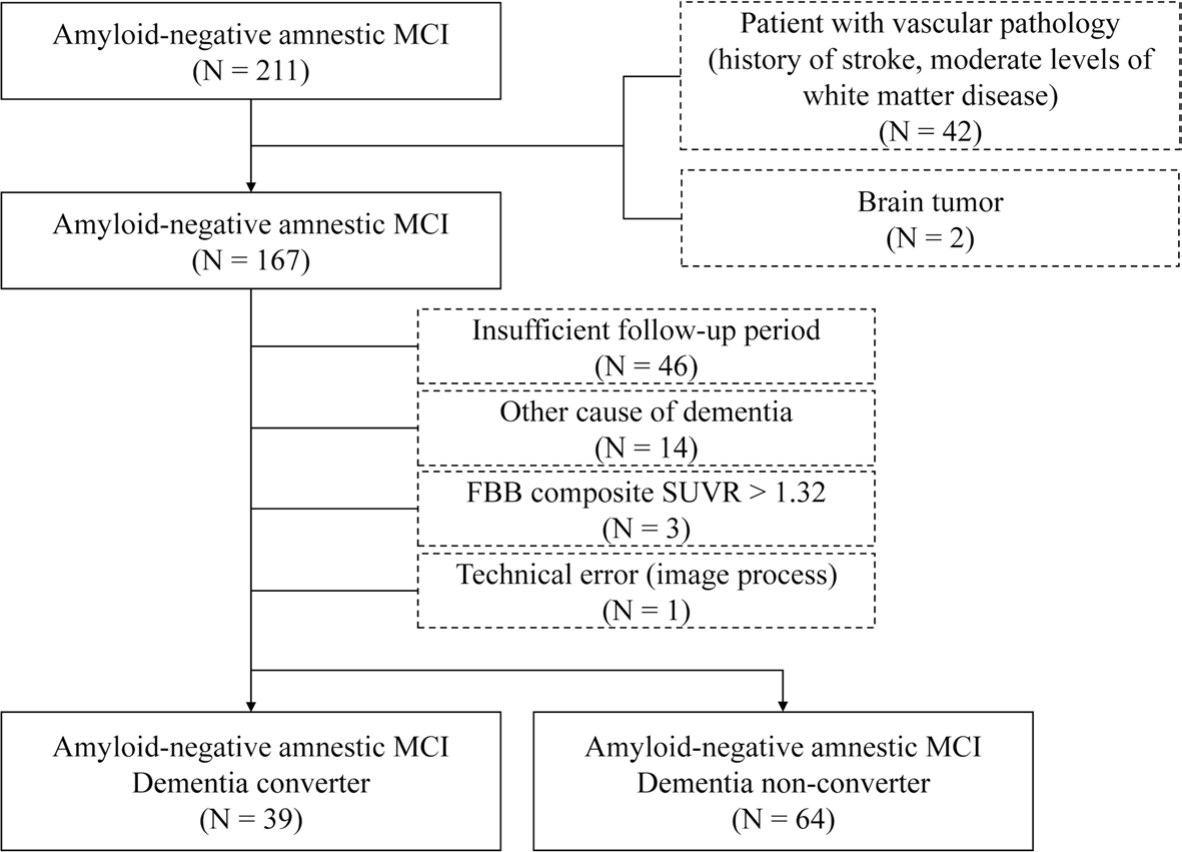
Flow chart for this study from the initial screening to the final analysis. The solid outline squares represent the subjects that remained. The dash line squares represent the excluded subjects. MCI, mild cognitive impairment; FBB, florbetaben

Patients with an intracranial hemorrhage, subdural hemorrhage, acute cerebral infarction, brain tumor, or white matter changes greater than a modified Fazekas scale score of 2 were excluded from the dataset (*N* = 42). Patients with a history of traumatic brain injury, seizure, brain surgery, or a current systemic medical disease were excluded (*N* = 2). Other causes of dementia, such as Parkinson’s disease, corticobasal syndrome, diffuse Lewy body dementia, idiopathic normal hydrocephalus, and frontotemporal degeneration, were excluded (*N* = 14). Patients who met the Diagnostic and Statistical Manual of Mental Disorders (fourth edition) criteria for psychotic or mood disorders were also excluded from the dataset. In total, 46 subjects whose follow-up period did not meet the 36-month timeline were excluded from the dataset. All evaluation processes were performed around 3 months based on the time when the neuropsychological test (Seoul Neuropsychological Screening Battery [SNSB]) was performed. Of the 107 patients who met the aforementioned criteria, four were excluded because of errors during image processing (one patient) or had an FBB composite score above 1.32 (three patients); therefore, the final sample set consisted of 103 patients with amyloid-negative amnestic MCI (Fig. 1).

Of the 103 patients with amyloid-negative amnestic MCI, conversion to dementia occurred in 39 (38%) patients at 36 months [18]. All patients visited the memory clinic regularly at intervals of 2 to 6 months and were interviewed by neurologists. The point of conversion was determined by a clinical interview by a skilled neurologist and neuropsychological tests including the Seoul-Instrumental Activities of Daily Living Scale. For patients who did not undergo a detailed neuropsychological test (*n* = 19), an experienced neurologist (J.H.L, H.J.K, and S.J.L) determined their conversion based on a decline in the Korean version of the Mini-Mental State Examination (MMSE) scores of more than 4 points per year with evidence of dysfunction in instrumental ADL (use of public transportation, shopping independently, and banking) [29–31].

To increase the reliability of the results of the study, we analyzed the Alzheimer’s Disease Neuroimaging Initiative (ADNI) dataset together. The data used in the preparation of this article were obtained from the Alzheimer’s Disease Neuroimaging Initiative (ADNI) database (adni.loni.usc.edu). The ADNI was launched in 2003 as a public-private partnership, led by principal investigator Michael W. Weiner, MD. The primary goal of ADNI has been to test whether serial magnetic resonance imaging (MRI), positron emission tomography (PET), and clinical and neuropsychological assessment can be combined to measure the progression of mild cognitive impairment (MCI) and early Alzheimer’s disease (AD). For up-to-date information, see www.adni-info.org. The UC Berkely cohort was selected because amyloid PET was performed with florbetaben tracer (UCBERKELEYFBB_04_26_22). The amyloid status was determined by a cutoff value of 1.08 (Composite SUVR) proposed for cross-sectional studies. The inclusion criteria for subjects were as follows: (1) initial diagnosis was confirmed as MCI and (2) at least 24 years of follow-up period. The disease progression was determined based on a decline in MMSE score more than 4 points or increased CDR score more than 0.5 points per year. Of 358 participants who had available florbetaben scans, 213 subjects were confirmed amyloid-negative status. Next step, 132 subjects with cognitive normal and 8 subjects with dementia were excluded from the dataset. The incomplete follow-up period subjects were composed of participants with no cognitive function test results other than the initial visit (*N* = 16; all subjects were MCI), or subjects with no diagnosis were made (*N* = 9). Finally, 48 subjects had included in the final dataset with 11 progressors and 37 non-progressors (Fig. S1).

### Cognitive assessment

All patients were assessed using the SNSB as a formal test, the details of which are described in Additional file 1. We also performed other clinical and cognitive performance measurements, including the Korean version of the Mini-Mental State Examination (K-MMSE), Global Deterioration Scale, Clinical Dementia Rating (CDR), the Korean version of the Neuropsychiatric Inventory, and the 30-item Geriatric Depression Scale (30-GDS) [32].

### Imaging acquisition

MRI was performed with a 3.0-T system (Achieva, Philips Medical Systems; Best, the Netherlands) using a sensitivity-encoding eight-channel head coil. A high-resolution anatomical three-dimensional (3D) volume image was obtained using a 3D gradient-echo T1-weighted sequence with the following parameters: repetition time/echo time, 9.9/4.6 ms; flip angle, 8°; field of view, 224 × 224 mm; matrix, 224 × 224; and slice thickness, 1 mm with no gap. According to the inclusion and exclusion criteria, the T1 magnetization-prepared rapid gradient-echo, fluid-attenuated inversion recovery, and T2 scans were reviewed to exclude the presence of structural lesions, which could rapidly aggravate cognitive functions.

All FBB PET images were obtained using Discovery 690, 710, and 690 Elite PET/computed tomography scanners (GE Healthcare; Chicago, IL). Amyloid PET images were acquired for 20 min, beginning 90 min after an injection of 300 ± 30 MBq ^18^F-florbetaben.

### Florbetaben PET processing and calculation of SUVR

Pre-processing of MRI data were made using the freely available software FreeSurfer (version 6.0; http://surfer.nmr.mgh.harvard.edu). This processing included motion correction, removal of non-brain tissue using a hybrid watershed/surface deformation procedure, automated Talairach transformation, intensity normalization, tessellation of the gray/white matter boundary, automated topology correction, and surface deformation following intensity gradients to optimally place the gray/white and gray/cerebrospinal fluid borders at the location where the greatest shift in intensity defined the transition to the other tissue class e. In these processes, segmentation and parcellation of T1-weighted images were conducted by automatically [33].

The volume of interest was defined on a stereotaxic atlas, with a corresponding template. In this study, the template image is non-linearly coregistered to the T1 MR-parcellated space and subsequently aligned to the native PET space to the subjects [33, 34].

Aβ burden was assessed using FBB PET. FBB PET data were expressed as SUVR scaled on a composite reference region including the whole cerebellum [35, 36]. VOIs were individually defined in the bilateral frontal, temporal, parietal, and occipital cortices; bilateral posterior cingulate and precuneus; bilateral striatum and thalamus; and brainstem and cerebellar cortex. Standardized uptake values for the regional VOIs were obtained, and regional SUVRs were calculated using the whole cerebellum as a reference region [27].

In the case of the ADNI dataset, we used SUVRs of each VOIs already presented. VOIs individually defined in bilateral frontal, temporal, parietal, posterior cingulate, and precuneus.

### Statistics

Data were analyzed using the Student *t*-test, Kruskal-Wallis test, chi-square test, analysis of covariance (ANCOVA), and time-dependent receiver operating characteristic (ROC) curve analysis. We divided patients into two groups, and no post hoc analyses were performed.

To compare the demographic profiles of the two groups (dementia non-converters, and dementia converters), we used a Student *t*-test for normally distributed data. For continuous variables that did not show normal distributions, the Kruskal-Wallis test was performed. To evaluate the group differences in dichotomous variables, we used the chi-square test.

For the comparisons of the regional amyloid burden by quantitative SUVRs, we used ANCOVA. The age and total cerebral gray matter volume (eTIV) were adjusted [37]. To determine the influence of regional amyloid burden to conversion to dementia from amnestic MCI, we used time-dependent ROC curve analysis with the VOIs showing the group differences identified by the ANCOVA test. Since the SUVR values of each VOI showed high multicollinearity, a regression model was deemed unsuitable. Therefore, we tried to confirm the biological meaning through an area under the curve (AUC) analysis of the time-dependent ROC curve. VOIs for AUC analysis were determined through the stepwise backward elimination process. Delong’s test was further carried out to confirm the high-risk model of conversion to dementia between the two most powerful models among the combination of VOIs.

To evaluate the association between baseline neuropsychologic profiles (age-adjusted *Z*-scores of each cognitive domain) and regional amyloid uptake, Spearman’s correlation analyses were used. We performed the *Z*-test to confirm that the difference in the correlation coefficient between the two groups was statistically significant. The significance level was determined to be 0.05.

$$Z=\frac{z_{r_1}-{z}_{r2}}{\sqrt{\frac{1}{n_1-3}+\frac{1}{n_2-3}}}$$

where *r*_1_ refers Pearson’s correlation coefficient of the converter group and *r*_2_ refers Pearson’s correlation coefficient of the non-converter group.

All statistical analyses were performed using R v4.0.3 (Institute for Statistics and Mathematics, Vienna, Austria; www.R-project.org), which was also used to derive the estimates of 95% of confidence intervals and standard error.

## Results

### Characteristics and demographics

The characteristics and demographics of the participants are displayed in Table 1. The age at onset of the converter group was greater than that of the non-converter group (*P* = 0.036). Additionally, females were 2.5 times more likely than males to convert to dementia (*P* = 0.038). However, neither vascular risk factors (hypertension, diabetes mellitus, and hyperlipidemia) nor the number of ApoE ε4 carriers was significantly different between the groups. Notably, significantly poorer performances on the K-MMSE and CDR sum of boxes (K-MMSE − 2.5 ± 0.8, *P* < 0.001; CDR sum of boxes 1.02 ± 0.20, *P* < 0.001) were observed in the converter group.

**Table 1:** Demographics and baseline characteristics of the patients

	Non-converter (*n* = 64)	Converter (*n* = 39)
Age of onset (years)*	73.0 ± 6.8	76.0 ± 0.0
Age at diagnosis (years)	72.0 ± 8.8	74.6 ± 6.7
Duration from onset to diagnosis (months)	32.0 ± 29.5	27.6 ± 21.3
Education (months)	10.2 ± 5.7	9.2 ± 5.5
Sex (female)*	32 (50.0%)	11 (28.2%)
Vascular risk factor		
Diabetes	20 (31.3%)	13 (33.3%)
HTN	36 (56.3%)	24 (61.5%)
ApoE genotype (carrier, %)	11 (20.0%)	7 (14.2%)
Global cognition test		
MMSE*	26.3 ± 3.7	23.5 ± 4.1
CDR Sum of Box*	1.4 ± 0.7	2.4 ± 1.2
30-GDS	13.6 ± 8.3	12.8 ± 6.4

The Student *t*-test was performed on normally distributed data. For continuous variables that did not show normal distributions, the Kruskal-Wallis test was performed. Group differences in dichotomous variables were evaluated using the χ^2^ tes*t*

*HTN*, hypertension; *MMSE*, Mini-Mental State Examination; *CDR*, Clinical Dementia Rating; *30-GDS*, 30-item Geriatric Depression Scale

*P < 0.05

### Difference in amyloid deposition between converters and non-converters

The differences between the two groups in terms of regional SUVRs that covariated with diagnosed age and total cerebral gray matter volume are displayed in Table 2. The converter group displayed increased regional SUVRs in the left temporal (converter 1.0091 ± 0.0969; non-converter 0.9589 ± 0.0832; *P* = 0.006), right temporal (converter 1.0048 ± 0.1080; non-converter 0.9645 ± 0.0786; *P* = 0.047), left parietal (converter 1.0847 ± 0.1060; non-converter 1.0080 ± 0.1213; *P* = 0.001), right parietal (converter 1.0730 ± 0.1092; non-converter 1.0080 ± 0.0730; *P* = 0.007), left precuneus (converter 1.1365 ± 0.1568; non-converter 1.0470 ± 0.1461; *P* = 0.007), left posterior cingulate (converter 1.0434 ± 0.0986; non-converter 0.9933 ± 0.1083; *P* = 0.021), right posterior cingulate (converter 1.0373 ± 0.1044; non-converter 0.9876 ± 0.1107; *P* = 0.026), left occipital (converter 1.1108 ± 0.1201; non-converter 1.0650 ± 0.1059; *P* = 0.046), and right occipital (converter 1.1161 ± 0.1123; non-converter 0.0501 ± 0.1021; *P* = 0.003) cortices. However, the largest difference was observed in the FBB composite SUVR (converter 1.1248 ± 0.1500; non-converter 1.0509 ± 0.1448; *P* = 0.015).

**Table 2:** Differences in regional SUVR between the converter and the non-converter groups

	Non-converter (*N* = 64)	Converter (*N* = 39)	*P*
L) Frontal cortex	0.9785 ± 0.1392	1.0123 ± 0.1406	0.238
R) Frontal cortex	0.9784 ± 0.1350	1.0182 ± 0.1558	0.177
L) Middle frontal cortex	0.9833 ± 0.1430	1.0313 ± 0.1615	0.124
R) Middle frontal cortex	0.9838 ± 0.1397	1.0345 ± 0.1802	0.117
L) Temporal cortex*	0.9589 ± 0.0832	1.0091 ± 0.0969	0.010
R) Temporal cortex*	0.9645 ± 0.0786	1.0048 ± 0.1080	0.036
L) Parietal cortex**	1.0025 ± 0.1213	1.0847 ± 0.1060	0.002
R) Parietal cortex*	1.0080 ± 0.1213	1.0730 ± 0.1092	0.011
L) Precuneus**	1.0470 ± 0.1461	1.1365 ± 0.1568	0.007
R) Precuneus	1.0610 ± 0.1405	1.1125 ± 0.1301	0.074
L) Cingulate cortex	1.0436 ± 0.1447	1.0780 ± 0.1548	0.258
R) Cingulate cortex	1.0400 ± 0.1454	1.0652 ± 0.1253	0.372
L) Striatum	1.1882 ± 0.0814	1.2057 ± 0.1643	0.470
R) Striatum	1.1904 ± 0.0858	1.2122 ± 0.1476	0.343
L) Posterior cingulate	0.9933 ± 0.1083	1.0434 ± 0.0986	0.306
R) Posterior cingulate	0.9876 ± 0.1107	1.0373 ± 0.1044	0.413
L) Occipital cortex**	1.0650 ± 0.1059	1.1108 ± 0.1201	0.005
R) Occipital cortex	1.0501 ± 0.1021	1.1161 ± 0.1123	0.050
FBB composite (SUVR)*	1.0508 ± 0.1448	1.1248 ± 0.1500	0.019

The differences in the regional SUVR between the two groups were analyzed using the analysis of covariance (ANCOVA). The data was adjusted to account for age at diagnosis and total cerebral gray matter volume

*FBB*, florbetaben; *SUVR*, standardized uptake value ratio

*P < 0.05

**P < 0.01

### Conversion to dementia model

**Fig. 2:**
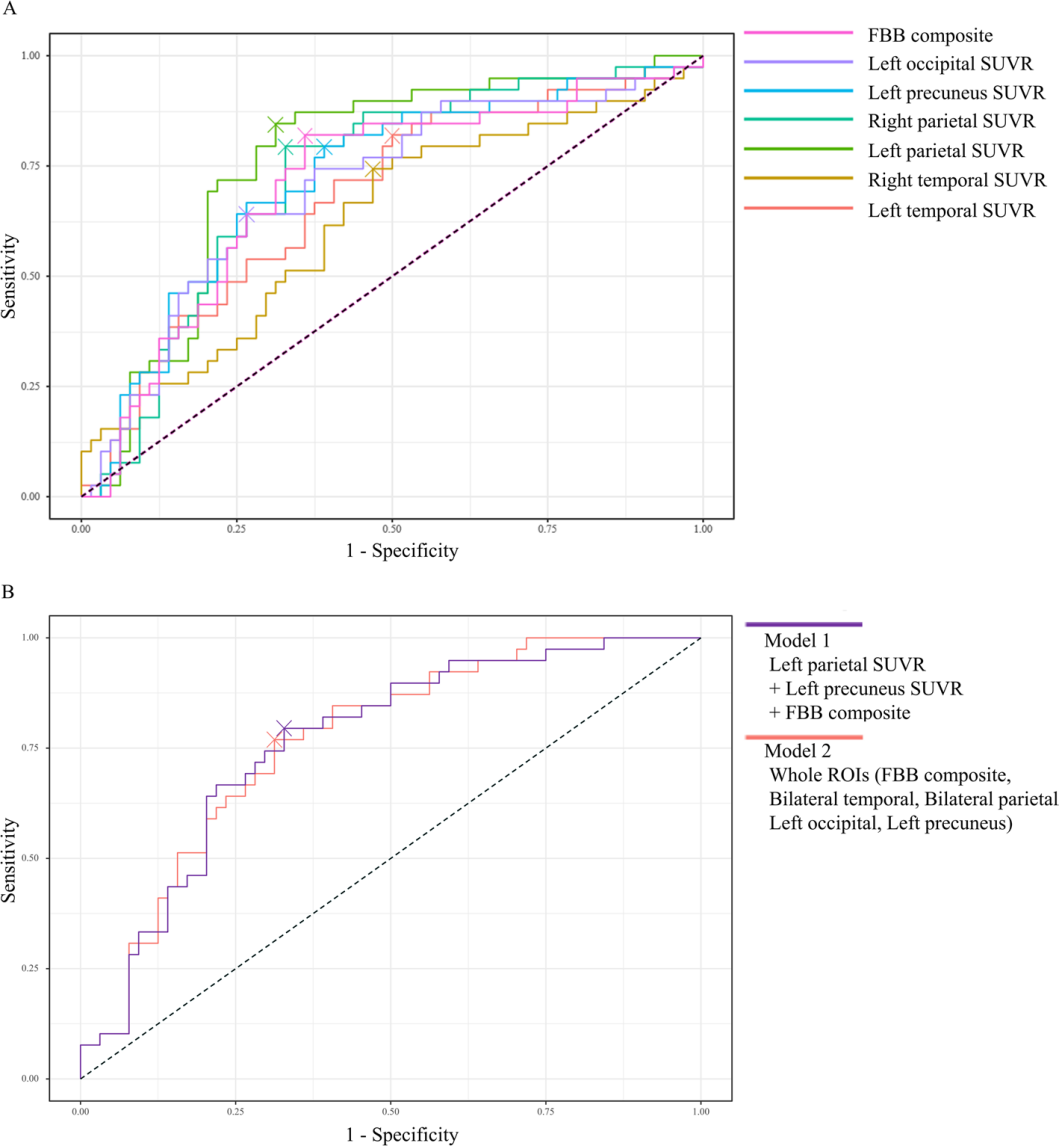
ROC comparison of dementia conversion model. **A** Results of ROC curve analysis. All VOIs that had group differences between converters and non-converters were related with dementia conversion. Bilateral parietal cortices showed high AUC compared to other VOIs. **B** Two models were selected for comparison. Model 1 included three VOIs, and model 2 included whole VOIs. There were no significant differences between the two models in distinguishing converters and non-converters. ROC, receiver operating characteristic; AUC, area under the curve; SUVR, standardized uptake value ratio; VOIs, volume of interests; FBB, florbetaben

The results from the ROC curve analysis revealed that all VOIs that had group differences were related to conversion to dementia (Table 3). Among all the VOIs, the SUVR of the left parietal cortex showed the highest AUC in conversion to dementia (SUVR cutoff value, 1.00438; AUC = 0.762; *P* < 0.001). Additionally, the ROC curve showed that the AUCs of the left precuneus and the right parietal cortex were higher than the AUCs of the bilateral temporal cortices (Fig. 2A).

**Table 3:** Influence of regional amyloid deposition on conversion to dementia

	Cutoff SUVR	AUC	Sensitivity (%)	Specificity (%)	*P* value	DeLong’s test *P* value
L) Temporal cortex	0.94203	0.678 (0.571–0.786)	82.1	50.0	< 0.001	
R) Temporal cortex	0.94333	0.624 (0.51–0.737)	74.4	53.1	< 0.001	
L) Parietal cortex	1.00438	0.762 (0.665–0.859)	84.6	68.8	< 0.001	
R) Parietal cortex	1.00589	0.724 (0.623–0.825)	79.5	67.2	< 0.001	
L) Precuneus	1.02879	0.721 (0.617–0.824)	79.5	60.9	< 0.001	
L) Occipital cortex	1.07716	0.703 (0.596–0.809)	64.1	73.4	< 0.001	
FBB composite	1.03224	0.708 (0.601–0.814)	82.1	64.1	< 0.001	
DeLong’s test for the two correlated ROC curves						
**Model 1**						
Left parietal SUVR + left precuneus SUVR + FBB composite		0.763 (0.67–0.856)	79.5	67.2	0.002	0.862
**Model 2**						
Whole VOIs (bilateral temporal, bilateral parietal, left precuneus, left occipital, FBB composite)		0.765 (0.673–0.857)	76.9	68.8	0.002

We selected two models that could predict conversion to dementia. Model 1 was composed of three VOIs (left parietal cortex SUVR, left precuneus cortex SUVR, and FBB composite), and model 2 was composed of whole VOIs (bilateral temporal, bilateral parietal, left precuneus, left occipital, and FBB composite). The result of stepwise backward elimination was displayed in Additional file 1: Table S1. Comparing model 1 with model 2, there was no significant difference in the prediction of conversion to dementia (DeLong’s test, *P* = 0.862, *Z* = 0.174).

The results from the ROC curve analysis of the ADNI dataset was displayed in Table 4. Among all VOIs, the SUVR of the left precuneus showed the highest AUC in the prediction of disease progression (SUVR cutoff value, 1.244; AUC = 0.596, *P* = 0.006). We selected two models that could predict conversion to dementia. Model 1 was composed of two VOIs (left precuneus, and bilateral frontal after the stepwise elimination process), and model 2 was composed of whole VOIs (bilateral frontal, temporal, parietal, precuneus, posterior cingulate, and FBB composite). Comparing model 1 with model 2, there was no significant difference in the prediction of conversion to dementia (DeLong’s test, *P* = 0.264, *Z* = 1.116).

**Table 4:** Influence of regional amyloid deposition on conversion to dementia (ADNI dataset)

	Cutoff SUVR	AUC	Sensitivity (%)	Specificity (%)	*P* value	DeLong’s test *P* value
Bilateral frontal	1.1726	0.582 (0.412–0.753)	63.6	52.8	0.006	
Bilateral parietal	1.2031	0.523 (0.348–0.698)	54.5	50.0	0.006	
Bilateral temporal	1.1257	0.523 (0.348–0.698)	54.5	50.0	0.006	
L) Precuneus	1.244	0.596 (0.426–0.766)	63.6	55.6	0.006	
R) Precuneus	1.2433	0.537 (0.362–0.712)	54.5	52.8	0.006	
L) Posterior cingulate	1.3054	0.537 (0.362–0.712)	54.5	52.8	0.006	
R) Posterior cingulate	1.3244	0.537 (0.362–0.712)	54.5	52.8	0.006	
FBB composite	1.1759	0.582 (0.412–0.753)	52.8	63.6	0.006	
DeLong’s test for the two correlated ROC curves						
**Model 1**						
Bilateral frontal, left precuneus		0.664 (0.487–0.841)	27.3	94.4	< 0.001	0.293
**Model 2 (whole VOIs)**						
Bilateral frontal, temporal, parietal, posterior cingulate, precuneus, FBB composite		0.732 (0.567–0.893)	72.7	61.1	<0.001	

To reveal the factors that influence the conversion to dementia, a ROC curve analysis was performed, and DeLong’s test was performed to compare the two most significant ROC models of conversion to dementia

*SUVR*, standardized uptake value ratio; *AUC*, area under the curve; *R*, right; *L*, left; *FBB*, florbetaben

To reveal the factors that influence the disease progression, a ROC curve analysis was performed, and eLong’s test was performed to compare the two most significant ROC models of conversion to dementia

*SUVR*, standardized uptake value ratio; *AUC*, area under the curve; *R*, right; *L*, left; *FBB*, florbetaben

### Neuropsychologic tests and regional amyloid burden

The results of the baseline neuropsychological tests (SNSB) for the two groups are displayed in Table 5. In the converter group, significantly worse performance was observed in the visual memory tasks compared to the non-converter group (Rey complex figure test [RCFT]-immediate, *P* = 0.001; RCFT-delayed recall, *P* < 0.001; RCFT-recognition, *P* = 0.001). The converter group also displayed a significantly lower controlled oral word association test animal task score than the non-converter group (*P* = 0.018).

**Table 5:** Results of the neuropsychologic test between the two groups

		Non-converter (***N*** = 64)	Converter (***N*** = 39)	***P*** value
**Attention**	Digit span forward	− 0.223 ± 1.238	0.033 ± 1.140	0.300
	Digit span backward	− 0.391 ± 1.175	− 0.251 ± 1.262	0.579
**Language**	K-BNT	− 0.637 ± 1.078	− 1.062 ± 1.229	0.069
**Visuospatial**	RCFT copy	0.006 ± 0.937	− 0.469 ± 1.442	0.072
**Memory**	SVLT-E immediate recall	− 1.103 ± 0.771	− 1.071 ± 0.855	0.842
	SVLT-E delayed recall	− 1.268 ± 0.925	− 1.610 ± 0.834	0.063
	SVLT-E recognition	− 0.909 ± 1.153	− 0.971 ± 1.203	0.795
	RCFT immediate recall*	− 0.563 ± 0.952	− 1.120 ± 0.679	0.001
	RCFT delayed recall*	− 0.652 ± 0.854	− 1.321 ± 0.648	< 0.001
	RCFT recognition*	− 0.571 ± 1.393	− 1.355 ± 0.996	0.001
**Frontal/executive**	COWAT animal*	− 0.346 ± 1.563	− 0.970 ± 1.045	0.018
	COWAT supermarket	− 0.441 ± 1.210	− 0.808 ± 0.698	0.056
	COWAT phonemic	− 0.613 ± 1.064	− 0.825 ± 0.755	0.273
	Stroop test color reading	− 0.872 ± 1.402	− 1.222 ± 1.163	0.214

The results of the neuropsychological tests were analyzed using an age-adjusted ANCOVA test

*K-BNT*, Korean version Boston naming test; *RCFT*, Rey complex figure test; *SVLT-E*, Seoul verbal learning test-elderly; *COWAT*, controlled oral word association test; *ANCOVA*, analysis of covariance

*P < 0.05

**Fig. 3.**
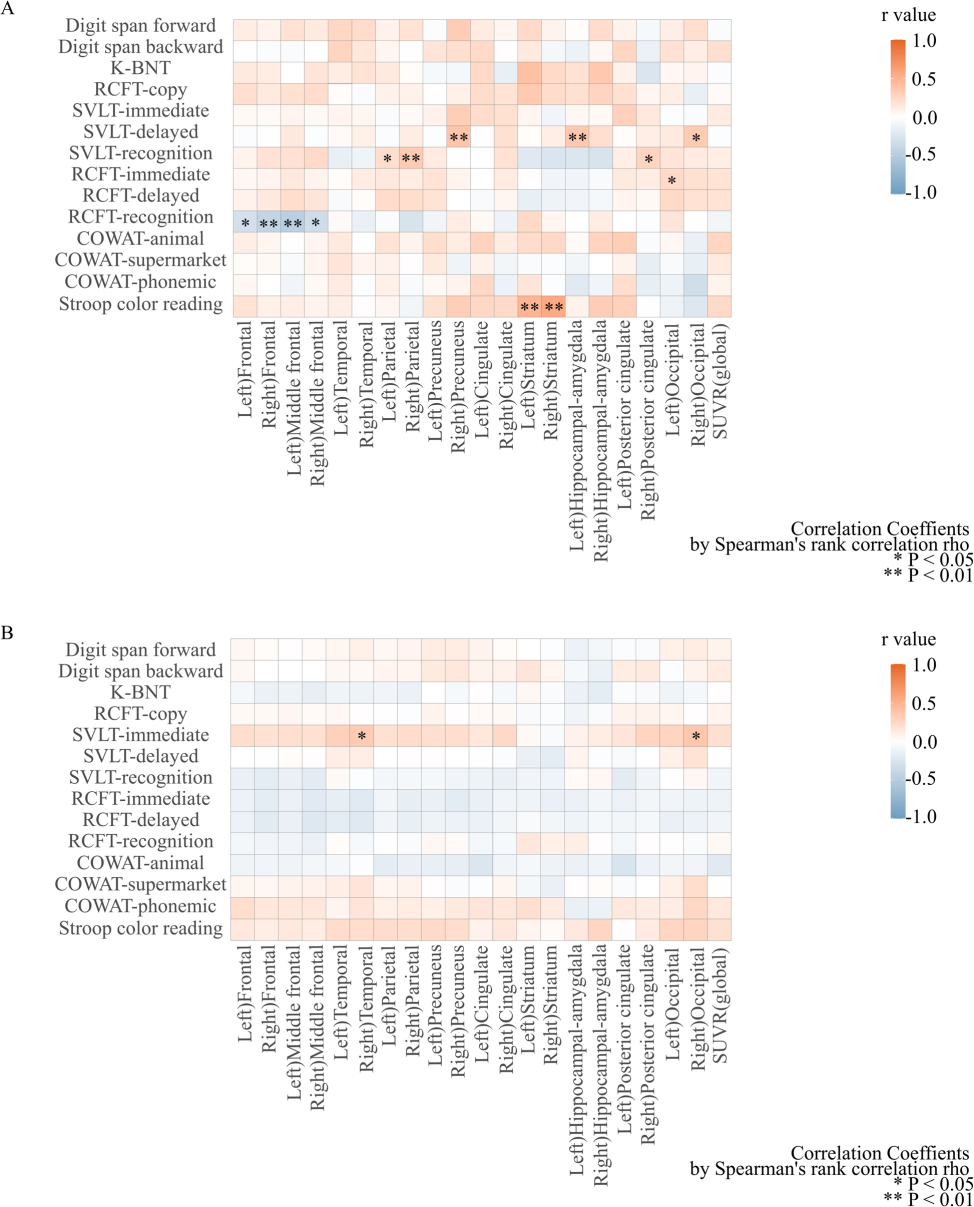
Correlation between neuropsychologic test performance and regional amyloid deposition. **A** Correlation coefficients between neuropsychologic test performance and regional amyloid deposition in the converter group. The converter group showed an inverse correlation between the score of the RCFT recognition and the SUVR in the bilateral frontal cortices. Interestingly, the score on the Stroop color reading test, which represents the frontal function, was positively correlated with bilateral striatum amyloid deposition. A Spearman correlation test was performed. **B** Correlation coefficients between neuropsychological test performance and regional amyloid deposition in the non-converter group. No significant correlations were observed. K-BNT, Korean version-Boston naming test; RCFT, Rey complex figure test; SVLT, Seoul verbal naming test; COWAT, controlled oral word association test; SUVR, standard uptake value ratio. *P < 0.05; **P < 0.01

The correlation between the results of each cognitive domain score in the baseline neuropsychologic test and the regional amyloid deposition revealed different patterns between the converter and the non-converter groups. In the converter group, their RCFT-recognition score was negatively correlated with the SUVR in bilateral frontal and middle frontal cortices (left frontal SUVRs, rho = − 0.30, *P* = 0.049; right frontal SUVRs, rho = − 0.42, *P* = 0.008; left middle frontal SUVRs, rho = − 0.48, *P* = 0.002; right middle frontal SUVRs, rho = − 0.34, *P* = 0.037; Spearman’s correlation). Additionally, their scores on the Stroop color reading test were positively correlated with the SUVR in the bilateral striatum (left striatum, rho = 0.49, *P* = 0.003; right striatum, rho = 0.56, *P* < 0.001) (Fig. 3A). In the non-converter group, no specific correlation was observed except a weak positive correlation with the Seoul verbal learning test-immediate recall task and the SUVRs of the right temporal cortex and the right occipital cortex (Fig. 3B). As a result of the *Z*-test, the correlation observed in the converter group was statistically significant except correlation between right middle frontal SUVR and RCFT-immediate recall scores and right occipital SUVR and SVLT-delayed recall scores. However, there was no statistical significance observed in the non-converter group (Additional file 2: Table S2).

## Discussion

In this amyloid-negative amnestic MCI cohort study, three main findings emerged in terms of conversion to dementia. First, the FBB SUVRs of the converter group were higher than those of the non-converter group in the temporal, parietal, and posterior cingulate cortices including the FBB composite score. Second, the SUVR of the parietal cortices predicted dementia conversion with the highest accuracy in this amnestic MCI with visually negative FBB PET scan compared to other VOIs. In particular, the combination of parietal, precuneus, and FBB composite score SUVRs predicted dementia conversion with the highest accuracy among our model comparisons. Third, although regional amyloid deposition is known to poorly correlate with neurological symptoms, we found that RCFT recognition test scores and regional amyloid burden of bilateral frontal cortices were negatively correlated in the converter group [38, 39]. Taken together, this suggests the potential value of using subthreshold levels of amyloid deposition as a marker for predicting conversion to dementia from amnestic MCI, even in patients with visually amyloid-negative MCI on PET.

We found that the global deposition of amyloid was higher in the converter group than in the non-converter group. In particular, we confirmed that higher regional SUVRs were observed in the converter group than in the non-converter group, which is similar to the tau deposition of AD continuum corresponding to Braak stages III–IV [40, 41]. Taken together, our findings suggest that subthreshold amyloid deposition plays a role in the conversion to dementia in two ways. First, subthreshold levels of amyloid deposition could induce pathological tau deposition in the brain. In fact, a previous study found that a low level or subthreshold level of Aβ was associated with deposition and spreading of pathologic tau protein even in a cognitively normal patient group [4, 5]. In particular, the subsequent tau deposition in Braak stage I–II regions was best predicted by the baseline Aβ burden [42–44]. Therefore, similar to the AD continuum, subthreshold Aβ deposition-induced pathologic tau deposition could be a causative factor of cognitive decline and conversion to dementia in these subpopulations [5]. In a previous study, we found out that the hippocampus volume of the converter group was reduced compared to the non-converter group [20]. In this study, the relationship between SUVR value and hippocampus volume was not found, suggesting the possibility of underlying tau pathology [45]. However, it is difficult to figure out the rationale for amyloid deposition and dementia conversion in terms of ATN classification in this population, except for the possibility of T marker directly impacting on neurodegeneration (N) in the absence of an overt A marker. A subtle increase in the subthreshold amyloid levels might affect the pathologic tau protein aggregation. Second, subthreshold levels of Aβ deposition could lower the threshold for developing symptoms of combined pathological factors [46]. Notably, as shown by Additional file 1, no statistically meaningful associations were observed between cortical thickness and SUVRs in either the converter or non-converter groups (Fig. S2). This suggests that there might be other pathological mechanisms beside amyloid that lead to neurodegeneration. For example, several studies have been conducted in vascular cognitive impairment (VCI) patients investigating the association between comorbid amyloid pathology and vascular burden, and shown that combined vascular and amyloid pathology revealed poor cognitive function in each case [47]. Furthermore, although still controversial, previous studies on VCI patients have found that patients with amyloid pathology had a poorer prognosis than those without amyloid pathology [14, 48, 49]. Thus, the small amount of Aβ deposition present in these patients could lower the threshold for the emergence of cognitive impairment caused by other pathology. In fact, other studies have reported that TDP-43 and hippocampal sclerosis are often associated in AD patients [50]. In particular, TDP-43 pathology is known to interact with amyloid deposition as well as pathological tau aggregation [51]. From this perspective, it is possible that TDP-43 pathology or hippocampal sclerosis might be related to amyloid pathology at the subthreshold levels, but future studies are needed to confirm this.

The second major finding of our study was that the regional amyloid burden in the parietal and precuneus predicted conversion to dementia with the highest accuracy. These results corroborate the previous findings showing that there is a relationship between amyloid accumulation and cognitive changes in amyloid-negative subjects using the ADNI data [7, 12]. Our results of analyzing ADNI dataset also revealed that the amyloid burden in the precuneus could predict cognitive decline in amyloid-negative MCI. Furthermore, even subthreshold levels of regional amyloid deposition could be prognostic factors in these patient groups. Other previous studies on the AD continuum have also revealed that the regional amyloid deposition in the parietal cortices and precuneus was important not only for cognitive decline, but also for conversion to dementia [52–54]. Notably, the effect of amyloid deposition has been shown to be the most robust in the precuneus and posterior cingulate cortices, key areas constituting the default mode network (DMN), in the AD continuum [55]. Interestingly, given our findings that the subthreshold levels of amyloid deposition in parietal cortices and precuneus are important for conversion to dementia, it is possible that these regions are responsible for cognitive decline. That is, these DMN-related regions may be dysregulated via amyloid deposition even at the subthreshold levels observed in our patient group [19, 56].

The third finding of our study was that performance on the visual memory retrieval task was correlated with the regional amyloid deposition in the bilateral frontal cortices, including the middle frontal cortex, only in the converter group [57]. Notably, there was no significant correlation between cognitive function and regional amyloid deposition in the non-converter group. In general, cognitive function along the AD continuum has been known to be more associated with the distribution of tau proteins than amyloid deposition [58–60]. In fact, it is questionable that frontal amyloid deposition alone causes pertinent cognitive deficit [61, 62]. Therefore, other pathologies such as pathological tau proteins or TDP-43 might be involved [16, 63]. Nevertheless, we did not observe a correlation between amyloid burden and quantitative structural brain MRI analysis. Future studies are warranted to further assess the effects of subthreshold levels of amyloid burden on cognitive function and brain structure.

### Limitations

There are some limitations in our study. First, the presence of other pathological proteins such as tau, TDP-43, and α-synuclein was not evaluated in this patient group [64]. Given the pathological heterogeneity and uncertainty of amyloid-negative amnestic MCI patients, postmortem pathological examination along with the active use of various biomarkers should be considered to elucidate the underlying pathophysiology of this condition. Other than VCI, few models of combined pathology interactions have been studied and presented yet. Also, because our dataset excluded patients with vascular pathology, there is a limitation in explaining our findings compared to VCI. Therefore, further research is warranted on the interaction of combined pathologies within our dataset. Second, we cannot completely exclude the possibility of a conversion to Alzheimer’s dementia from amyloid-negative MCI [12]. In other words, cumulating amyloid deposition might have gone above the threshold levels at some point in the course of time. Therefore, a follow-up PET scan should be performed to verify this. Finally, the follow-up neuropsychological assessments (SNSB) were not performed in all patients, which made it difficult to evaluate the changes in each cognitive domain. To overcome this, three experienced neurologists evaluated the patients according to the aforementioned criteria. However, due to the inherent problems with a retrospective study, it seems that selection bias could not be completely excluded from the process. The higher conversion to dementia rate of our patient group (36.4%) than other previous studies could also be due to selection bias. Future prospective studies, including longitudinal follow-up of structural and functional imaging with post-mortem pathological confirmations, are needed to better address the clinical progression of amyloid-negative MCI. Despite these limitations, our study assessed the effect of amyloid deposition on conversion to dementia in seemingly amyloid-negative MCI by excluding patients with an ischemic lesion and investigated the relationship between levels of regional amyloid uptake across each cognitive domain function by administering detailed neuropsychological tests.

## Conclusions

Our results suggest that the analysis of the regional Aβ burden, even at the subthreshold level, may provide significant information about dementia progression in patients with visually amyloid-negative MCI on PET.

## Supplementary Information

**Additional file 1: Table S1**. The result of stepwise backward elimination. **Figure S1**. Flow chart for this study of ADNI dataset. The solid outline squares represent subjects that remained. The dash line squares represent excluded subjects. Abbreviations: MCI, Mild cognitive impairment. **Figure S2**. Correlation between regional SUVR and cortical thickness in the converter group. (A) SUVR of the right middle frontal cortex and medial aspect of the cerebrum; (B) SUVR of the left hippocampus and medial aspect of the cerebrum; (C) SUVR of the right striatum lateral aspect of the cerebrum; (D) SUVR of the left occipital cortex lateral aspect of the cerebrum; (E) Right FBB composite and medial aspect of the cerebrum; (F) Left FBB composite and medial aspect of the cerebrum; (G) Right FBB composite and lateral aspect of the brain; and (H) Left FBB composite and lateral aspect of the brain. FDR correction with p < 0.05, and p value < 0.001. There was no statistical correlation in the non-converter group; thus, only the converter group’s results are shown from (A) to (G). SUVR, standard uptake value ratio; FBB, florbetaben; FDR, false discovery rate.

**Additional file 2: Table S2**. The results of Z-test (comparison of correlation coefficient) between the two group.

**Additional file 3.**

## Data Availability

The data are not publicly available due to privacy or ethical restrictions.

## Abbreviations

Aβ: β-Amyloid
ADL: Activities of daily living
AD: Alzheimer’s disease
ADNI: Alzheimer’s Disease Neuroimaging Initiative
AUC: Area under the curve
BAPL: Brain amyloid-plaque loading
CDR: Clinical Dementia Rating
DMN: Default mode network
FBB: ^18^F-florbetaben
K-MMSE: Korean version of the Mini-Mental State Examination
MCI: Mild cognitive impairment
PET: Positron emission tomography
ROC: Receiver operating characteristic
RCFT: Rey complex figure test
SNSB: Seoul Neuropsychological Screening Battery
SUVR: Standardized uptake value ratio
TDP-43: TAR-binding protein-43
VCI: Vascular cognitive impairment
VOIs: Volume of interests
