# Supplementary material for: The impact of subthreshold levels of amyloid deposition on conversion to dementia in patients with amyloid-negative amnestic mild cognitive impairment
Source: Alzheimers Res Ther. 2022 Jul 11;14:93. doi: 10.1186/s13195-022-01035-2 (PMC9277922; doi:10.1186/s13195-022-01035-2)
Supplement: Supplementary file 1 — Additional file 1: Table S1. The result of stepwise backward elimination. Figure S1. Flow chart for this study of ADNI dataset. The solid outline squares represent subjects that remained. The dash line squares represent excluded subjects. Abbreviations: MCI, Mild cognitive impairment. Figure S2. Correlation between regional SUVR and cortical thickness in the converter group. (A) SUVR of the right middle frontal cortex and medial aspect of the cerebrum; (B) SUVR of the left hippocampus and medial aspect of the cerebrum; (C) SUVR of the right striatum lateral aspect of the cerebrum; (D) SUVR of the left occipital cortex lateral aspect of the cerebrum; (E) Right FBB composite and medial aspect of the cerebrum; (F) Left FBB composite and medial aspect of the cerebrum; (G) Right FBB composite and lateral aspect of the brain; and (H) Left FBB composite and lateral aspect of the brain. FDR correction with p < 0.05, and p value < 0.001. There was no statistical correlation in the non-converter group; thus, only the converter group’s results are shown from (A) to (G). SUVR, standard uptake value ratio; FBB, florbetaben; FDR, false discovery rate. [file 13195_2022_1035_MOESM1_ESM.zip › Table S1.docx]

|  | Estimate | Standard error | Z-value | P |
| --- | --- | --- | --- | --- |
| (Intercept) | -6.44 | 2.31 | -2.78 | 0.005 |
| Left precuneus | 23.59 | 10.50 | 2.25 | 0.025 |
| Left parietal | 4.02 | 2.60 | 1.55 | 0.122 |
| FBB composite | -22.16 | 10.45 | -2.12 | 0.034 |
